# Additive effect of erythropoietin use on exercise-induced endothelial activation and hypercoagulability in athletes

**DOI:** 10.1007/s00421-020-04419-0

**Published:** 2020-06-14

**Authors:** Jules A. A. C. Heuberger, Jelle J. Posthuma, Dimitrios Ziagkos, Joris I. Rotmans, Johannes M. A. Daniels, Pim Gal, Frederik E. Stuurman, Henri M. H. Spronk, Hugo Ten Cate, Jacobus Burggraaf, Matthijs Moerland, Adam F. Cohen

**Affiliations:** 1 Centre for Human Drug Research, Zernikedreef 8, 2333 CL Leiden, The Netherlands; 2 Departments of Internal Medicine and Biochemistry, Cardiovascular Research Institute Maastricht (CARIM), Maastricht University, Maastricht, The Netherlands; 3 Department of Internal Medicine, Leiden University Medical Centre, Leiden, The Netherlands; 4 Department of Pulmonary Diseases, VU University Medical Centre, Amsterdam, The Netherlands; 5 Leiden Academic Centre for Drug Research, Leiden, The Netherlands

**Keywords:** Erythropoietin, Hemostasis, Exercise, Endothelial activation, Coagulation

## Abstract

**Purpose:**

Recombinant human erythropoietin (rHuEPO) is known to increase thrombotic risk in patients and might have similar effects in athletes abusing the drug. rHuEPO is prohibited by anti-doping legislation, but this risk has not been investigated thoroughly. This analysis was designed to evaluate whether rHuEPO impacts hemostatic profile and endothelial and platelet activation markers in trained subjects, and whether the combination with exercise affects exercise induced alterations.

**Methods:**

This double-blind, randomized, placebo-controlled trial enrolled healthy, trained male cyclists aged 18–50 years. Participants were randomly allocated (1:1) to receive subcutaneous injections of rHuEPO (epoetin-β; mean dose 6000 IU per week) or placebo (0.9% NaCl) for 8 weeks. Subjects performed five maximal exercise tests and a road race, coagulation and endothelial/platelet markers were measured at rest and directly after each exercise effort.

**Results:**

rHuEPO increased P-selectin (+ 7.8% (1.5–14.5), *p* = 0.02) and E-selectin (+ 8.6% (2.0–15.7), *p* = 0.01) levels at rest. Maximal exercise tests significantly influenced all measured coagulation and endothelial/platelet markers, and in the rHuEPO group maximal exercise tests led to 15.3% ((7.0–24.3%), *p* = 0.0004) higher E-selectin and 32.1% ((4.6–66.8%), *p* = 0.0207) higher Platelet factor 4 (PF4) levels compared to the placebo group.

**Conclusion:**

In conclusion, rHuEPO treatment resulted in elevated E- and P-selectin levels in trained cyclists, indicating enhanced endothelial activation and/or platelet reactivity. Exercise itself induces hypercoagulability, and the combination of rHuEPO and exercise increased E-selectin and PF4 levels more than either intervention alone. Based on this, exercise potentially increases thrombotic risk, a risk that might be enhanced in combination with rHuEPO use.

**Electronic supplementary material:**

The online version of this article (10.1007/s00421-020-04419-0) contains supplementary material, which is available to authorized users.

## Introduction

Recombinant human erythropoietin (rHuEPO) is on the World Anti-Doping Agency’s Prohibited List as it is considered to possess performance enhancing properties and represents a potential health risk to athletes (World Anti-Doping Agency 2018). Effects on actual performance, however, are not indisputably proven in athletes (Heuberger et al. 2013). Moreover, in a recent study we found that although rHuEPO treatment improved laboratory tests of maximal exercise, more relevant endurance performance variables such as time trial and road race performance remained unchanged (Heuberger et al. 2017). Similarly, although there is evidence that rHuEPO increases thrombogenicity and risk of stroke in patients (Pfeffer et al. 2009), it is unclear whether athletes abusing rHuEPO could also display increased thrombotic risk. It would be informative to understand whether rHuEPO activates coagulation and endothelial cells in athletes, especially given that exercise itself is known to impact the hemostatic profile (Posthuma et al. 2015). Although exercise is generally accepted to be beneficial for health, cases of thrombotic events induced by exercise have been reported. This is likely due to the effects of exercise on the hemostatic profile, which seem to be correlated with exercise intensity (Posthuma et al. 2015). Intense exercise, often performed by athletes, in combination with increased erythropoiesis and subsequent hemoconcentration after rHuEPO use might increase the risk for thrombotic events. Additionally, potential direct effects of rHuEPO on pathways involved in coagulation and endothelial and platelet activation could add to this risk. In the current analysis as part of the same study (Heuberger et al. 2017) we investigated the effects of rHuEPO, exercise and the combined effects of rHuEPO and exercise in trained cyclists on markers of coagulation and endothelial and platelet activation.

## Materials and methods

### Data sharing statement

The trial protocol can be found at:

http://chdr.nl/library/protocol-to-study-chdr1514-the-effects-of-erythropoietin-on-cycling-performance-of-well-trained-cyclists-a-randomized-double-blind-placebo-controlled-parallel-trial/download.

The data that support the findings of this study are available from the corresponding author upon reasonable request.

### Study design and participants

The study design was previously described (Heuberger et al. 2017). Briefly, forty-eight healthy male cyclists between 18 and 50 years participated in a double-blind, randomized, placebo controlled, parallel, single center study. Among the inclusion criteria were hemoglobin (Hb) concentration between 8.0 and 9.8 mmol/L (equivalent to 12.8–15.7 g/dL, within the normal range for this population) and hematocrit (Ht) level below 48% at screening and not using medication that could potentially interact with the study drugs or study assessments. Health status was determined by medical history and physical examinations; participants were excluded if they had any clinically relevant pre-existing condition, including cardiovascular, pulmonary, muscle, metabolic, hematological or inflammatory disease.

### Randomization and masking

Participants were randomly assigned in a balanced manner to either rHuEPO or placebo treatment. A stratified randomization block was used with participants aged 18**–**34 (inclusive) and aged 35**–**50 (inclusive) to reduce variability between groups due to age differences.

### Procedures

#### Treatment

Treatments were as described previously (Heuberger et al. 2017). Briefly, participants received weekly abdominal subcutaneous injections of Epoetin beta (NeoRecormon, Roche, Basel, Switzerland) prepared from multidose vials or saline (0.9% NaCl) for 8 weeks. Hb and Ht were measured before each dose and only available to dedicated un-blinded personnel. The target Hb in the rHuEPO group was an increase of 10–15% compared to the baseline Hb concentration, measured with the HemoCue Hb 201 + analyzer (Radiometer Benelux B.V., Zoetermeer, the Netherlands). In the first 4 weeks, participants received a 5000 international units (IU) dose per injection, after which it could be increased to 6000 IU, 8000 IU or 10000 IU to reach the target range. rHuEPO dose was 2000 IU if Hb was in the target range. For safety reasons, participants received a placebo injection if the Hb was above the target range, or if Ht was equal to or exceeded 52%, measured by the Hematokrit 200 (Hettich Benelux B.V., Geldermalsen, the Netherlands). rHuEPO and placebo were visually indistinguishable (both colorless solutions) and dosage changes (changes in injected volume) were also randomly assigned to placebo participants.

All participants also took open-label daily oral doses of 200 mg ferrous fumarate and 50 mg ascorbic acid (both from Pharmachemie B.V., Haarlem, The Netherlands) during the study to make sure participants were not iron deficient during the study.

#### Maximal graded exercise test

Maximal graded exercise tests were performed on a Monark LC4r ergometer (COSMED, Rome, Italy) during screening, at baseline (up to 14 days before first dose) and during the treatment period at 11, 25, 39 and 53 days (± 1 day) after the first dose administration. The protocol started with 1-min rest without pedaling, followed by 2 min warm-up at 75 Watts. The pedaling resistance was then increased to 175 Watts, and 25 watts every 5 min. Cadence had to be maintained between 70 and 90 rpm. The test stopped when cadence could not be maintained above 70 rpm or when a participant terminated the test. Participants then pedaled at 50 Watts during a 3-min recovery. Blood for coagulation and endothelial markers was drawn from an intravenous cannula in the right forearm before and directly after the exercise tests.

#### Mont ventoux race

Approximately 12 days after the last dose participants took part in a road stage of 110 km in the French Provence (total elevation gain 1524 m), after which they competitively climbed the Mont Ventoux (Vaucluse département, France) in an open course via Bédoin, France, bridging an altitude of 1610 m over 21.5 km. Blood for coagulation and endothelial markers was drawn before the 110 km stage and at the top of the Mont Ventoux.

#### Coagulation and endothelial and platelet markers

Markers for coagulation and platelet activation determined were prothrombin time (PT), activated partial thromboplastin time (aPTT), D-dimer, fibrinogen, Beta thromboglobulin (beta-TG), prothrombin fragment 1 + 2 (F1 + 2), Factor VIII (FVIII), platelet factor 4 (PF4) and Thrombin:Antithrombin (TAT). Endothelial activation markers determined were E-selectin, intercellular adhesion molecule 1 (iCAM), P-selectin, thrombomodulin, vascular cell adhesion molecule 1 (vCAM) and Von Willebrand Factor antigen (vWF). For more detail on the assays, see the Supplemental Material.

#### Blood sample handling quality control

Soluble CD40L is primarily produced and released by activated platelets. Numerous studies have reported increased sCD40L in various clinical conditions and diseases associated with platelet activation. When comparing two commercially available sCD40L ELISA kits (Thermo Fisher and RnD systems), all test samples collected in CTAD tubes showed sCD40L levels below or around the lower level of quantification. Further investigation indicated that when comparing CTAD and serum samples from the same donors, similar low sCD40L levels were detected in the CTAD samples, but significant sCD40L levels were present in the serum samples, demonstrating the efficacy of the used ELISA kits. These data indicate that careful sample handling procedures and collection in CTAD tubes resulted in minimal platelet activation and subsequent sCD40L release, and that minimal in vivo sCD40L release had taken place in the study participants.

### Statistical analysis

Participants with at least one available measurement were included in the analyses. For rHuEPO effects, pre-exercise data were analyzed with a mixed model analysis of variance with treatment, time and treatment by time as fixed factors, participants as random factor and the pre-value as covariate for treatment effects. For exercise effects, the analysis was performed on the placebo group, with a mixed model analysis of variance, time as fixed factor, participants as random factor and the pre-value as covariate. For combined rHuEPO and exercise effects, the analysis was performed on the post-exercise measures with a mixed model analysis of variance with treatment, time and treatment by time as fixed factors, participants as random factor and the pre-value as covariate for treatment effects. The contrast that is calculated within the models is placebo versus rHuEPO, or before versus after exercise.

Results of statistical models are reported as estimated means (EM) at the different time points per intervention and estimates of the difference between treatments over the whole time period, including 95% confidence intervals (log-transformed parameters are reported in percentage) and the *p* value of the contrasts.

When 95% confidence intervals are presented, they reflect the estimated difference between the two treatment groups, with a significance level of *p* < 0.050. All calculations were performed using SAS for windows V9.4 (SAS Institute, Inc., Cary, NC, USA).

## Results

### rHuEPO effects

A total of 48 participants were included in the analyses (24 in the rHuEPO group and 24 in the placebo group). One participant withdrew after the fourth dose administration, all other 47 participants completed the study. Participants were trained cyclist, confirmed by their average baseline maximal power output values of 335.07 W (SD 33.40) and maximal oxygen uptake (VO2max) of 55.63 mL/min per kg (SD 4.80), with average recorded cycling activity of 4.9 h and 5.9 h for rHuEPO and placebo groups, respectively. rHuEPO treatment had clear effects on several variables in rest as we have partly reported previously (Heuberger et al. 2017). Average Hb (+ 12%) and Ht (+ 16%) increased upon rHuEPO treatment and was significantly elevated compared to placebo with 0.6 mmol/L (0.44–0.77, *p* = < 0.0001) (equivalent to 0.97 g/dL) and 3.3% (2.5–4.1, *p* = <0.0001), respectively. In contrast, platelet count was not affected by rHuEPO treatment (see Supplemental Table 1). rHuEPO significantly increased resting levels of P-selectin (+ 7.8% (1.5–14.5), *p* = 0.02) and E-selectin (+ 8.6% (2.0–15.7), *p* = 0.01) compared to placebo, but iCAM and vCAM were not significantly altered by rHuEPO treatment, see Table 1. aPTT and coagulation markers TAT and D-dimer remained unchanged, as did specific platelet activation markers PF4 and Beta-TG, see Supplemental Table 1.

**Table 1:** Effects of rHuEPO on markers in rest

Parameter	Treatment	Raw baseline	EM Day 11	EM Day 14	EM Day 25	EM Day 28	EM Day 39	EM Day 42	EM Day 53	EM Pre-race	Difference between groups
E-selectin, pg/mL	Placebo	5100 (1978)	4986	4964	5097	5166	4731	4740	4942	4953	8.6% (2.0%, 15.7%) *p* = 0.011
	rHuEPO	5543 (1912)	5446	5117	5475	5270	5568	5251	5477	5387	
P-selectin, pg/mL	Placebo	8789 (2112)	9037	8714	8524	8930	8810	8721	8261	8599	7.8% (1.5%, 14.5%) *p* = 0.016
	rHuEPO	9710 (2285)	9010	9120	9248	9590	9846	9267	9173	9789	
iCAM, pg/mL	Placebo	318333 (48913)	320475	313774	321983	326577	317702	297252	306471	320147	2.2% (− 2.2%, 6.8%) *p* = 0.33
	rHuEPO	335958 (73686)	322643	322951	328572	330397	330440	310291	311309	322155	
vCAM, pg/mL	Placebo	489750 (84683)	527396	482540	506971	502660	498766	493731	514184	493716	− 0.0% (− 4.2%, 4.4%) *p* = 0.99
	rHuEPO	515000 (102348)	520718	480747	485750	496181	504203	508575	507573	515472	
Von Willebrand Factor, %	Placebo	88.4 (27.7)	81.953	86.168	85.932	91.320	86.046	88.634	83.627	90.113	− 0.9 (− 8.0%,6.8%) *p* = 0.8138
	rHuEPO	100.1 (25.4)	87.897	86.114	82.513	94.138	80.810	86.512	81.131	89.014	
PF4, pg/mL	Placebo	43019 (46073)	68339	23340	51235	28821	33209	35929	33026	63845	13.1% (− 9.3%, 41.1%) *p* = 0.2661
	rHuEPO	43988 (85138)	60195	23125	60555	31296	52786	36164	43549	72593	

Raw baseline (and SD) and EM (Estimated Mean) values of coagulation and endothelial activation markers in rest at the different time points for both treatment groups, including the estimated differences between the treatment groups (95% confidence interval) and *p* value. Data analyzed with a mixed model analysis of variance with fixed factors treatment, time and treatment by time, random factor participant and the pre-value as covariate

### Exercise effects

**Fig. 1:**
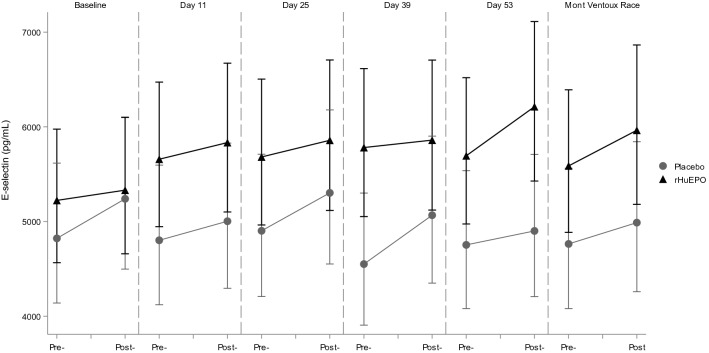
Exercise and rHuEPO effects on E-selectin levels (pg/mL). Pre- and post- exercise levels of E-selectin for the maximal graded exercise test at baseline, 11, 25, 39 and 53 days and the Mont Ventoux race for rHuEPO and placebo groups

**Fig. 2:**
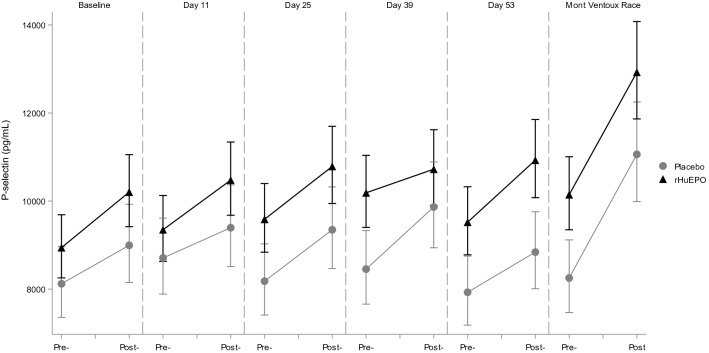
Exercise and rHuEPO effects on P-selectin levels (pg/mL). Pre- and post- exercise levels of P-selectin for the maximal graded exercise test at baseline, 11, 25, 39 and 53 days and the Mont Ventoux race for rHuEPO and placebo groups

**Fig. 3:**
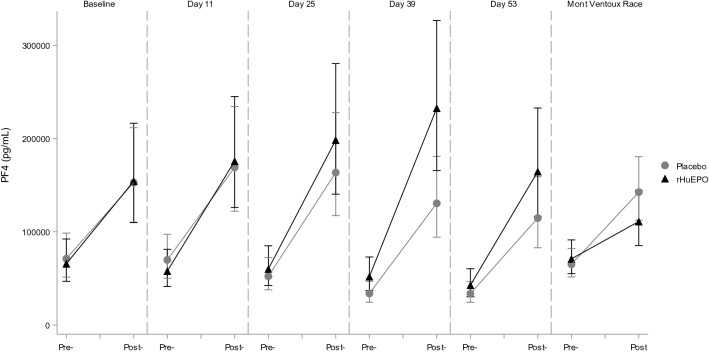
Exercise and rHuEPO effects on Platelet Factor 4 (pg/mL). Pre- and post- exercise levels of platelet factor 4 (PF4) for the maximal graded exercise test at baseline, 11, 25, 39 and 53 days and the Mont Ventoux race for rHuEPO and placebo groups

**Fig. 4:**
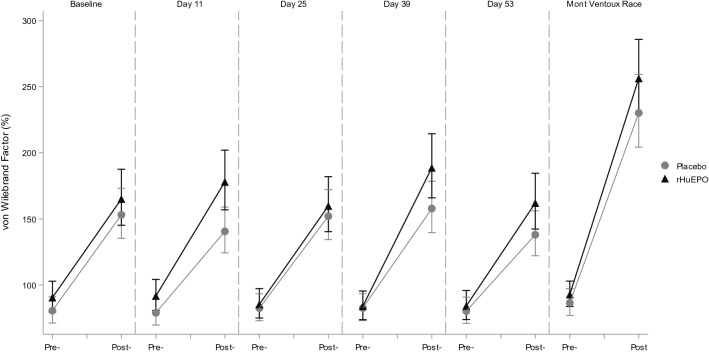
Exercise and rHuEPO effects on von Willebrand Factor (%). Pre- and post- exercise levels of von Willebrand Factor (vWF) for the maximal graded exercise test at baseline, 11, 25, 39 and 53 days and the Mont Ventoux race for rHuEPO and placebo groups

Exercise by itself induced significant alterations in hemostatic profile, as can be concluded from the observations in the placebo treated participants. Exercise increased TAT, D-Dimer, PF4 and Beta-TG levels, and decreased aPTT in this group. Endothelial markers E- and P-selectin were also increased by exercise, as well as iCAM and vCAM. All measured markers were significantly affected by the maximal exercise test, whereas the race only had a significant effect on a subset of markers. The direction of these significant effects was the same for the exercise test and race, although there seem to be differences in magnitude of the effect for some markers: aPTT (percentage change after maximal exercise test and race: − 12.4% and − 15.5%, respectively), Beta-TG (92.6% and 21.2%), FVIII (128.7% and 181.1%), P-selectin (12.2% and 34.1%), PF4 (192.4% and 118.9%), TAT (504.6% and 159.7%) and vWF (82.9% and 166.0%). See Table 2 for an overview of all changes in markers due to both the exercise test and the race and Figs. 1, 2, 3, 4 for a graphical representation of these effects for a selection of markers. Table 2 also shows a comparison of these observed effects with existing literature data. This comparison shows that the direction of the observed effect in the current study is supported by the data available, although the magnitude of the effect differs for some specific markers.

**Table 2:** Effects of different types of exercise on hemostatic profile: current study and literature data

Marker	Current study		Literature data		
	Exercise test	Race	Maximal exercise	Short (≤ 60 min) submaximal exercise	Long (> 60 min) submaximal exercise
Platelet count	NA	**10.6% [1.1%; 20.9%]**	**25 to 27%** (Herren et al. 1992; Wang et al. 1994)	26% (Cadroy et al. 2002)	12% (Posthuma et al. 2014)
Activated partial thromboplastin time	**− 12.4% [− 14.1%; − 10.6%]**	**− 15.5% [− 28.2%; − 0.5%]**	− **6%** (Menzel and Hilberg 2011; Handa et al. 1992)	− **5 to − 8%** (Handa et al. 1992; Menzel and Hilberg 2011)	NA
Prothrombin time	**− 2.1% [− 2.9%; − 1.3%]**	**1.5% [− 1.2%; 4.2%]**	− 1 to − 3% (Handa et al. 1992; Menzel and Hilberg 2011)	2% (Handa et al. 1992; Menzel and Hilberg 2011)	NA
Fibrinogen	**7.0% [4.4%; 9.6%]**	**− 7.6% [− 15.5%; 1.2%]**	**12%** (Gunga et al. 2002)	NA	2% (Rocker et al. 1986)
D-dimer	**43.0% [25.5%; 62.9%]**	**− 2.5% [− 24.3%; 25.6%]**	NA	~ **150%** (Parker et al. 2012)	NA
Beta Thromboglobulin	**92.6% [63.8%; 126.3%]**	21.2% [16.9%; 135.1%]	**60 to 85%** (Herren et al. 1992; Wang et al. 1994)	21% (Cadroy et al. 2002)	NA
E-selectin	**7.1% [4.3%; 9.9%]**	4.7% [− 16.1%; 30.7%]	4 to **10%** (Li et al. 2007; Jilma et al. 1997)	− 4% (Jilma et al. 1997)	**16%** (Nielsen and Lyberg 2004)
iCAM	**8.6% [6.5%; 10.9%]**	4.4% [-4.7%; 14.4%]	**11 to 24%** (Jilma et al. 1997; Rehman et al. 1997)	− 4% (Jilma et al. 1997)	**5%** (Nielsen and Lyberg 2004)
vCAM	**7.6% [5.7%; 9.4%]**	7.3% [− 2.3%; 17.9%]	10% (Jilma et al. 1997)	1% (Jilma et al. 1997)	**22%** (Nielsen and Lyberg 2004)
Prothrombin fragment 1 + 2	**88.0% [64.3%; 115.2%]**	**9.8% [− 12.6%; 38.1%]**	**17 to 19%** (Rocker et al. 1996; Li et al. 2007)	NA	NA
Factor VIII	**128.7% [104.4%; 155.9%]**	**181.1% [147.8%; 218.9%]**	**63%** (Menzel and Hilberg 2011)	**34 to 38%** (Hegde et al. 2001; Menzel and Hilberg 2011)	NA
P-selectin	**12.2% [9.1%; 15.4%]**	**34.1% [16.2%; 54.6%]**	**21%** (Li et al. 2007)	~ **70%** (Parker et al. 2012)	**44%** (Nielsen and Lyberg 2004)
PF4	**192.4% [137.2%; 260.6%]**	**118.9% [57.2%; 205.0%]**	**85%** (Wang et al. 1994)	NA	**102%** (Rocker et al. 1986)
Thrombin:Antithrombin	**504.6% [386.6%; 651.3%]**	**159.7% [65.2%; 308.5%]**	**32 to 108%** (Rocker et al. 1996; Menzel and Hilberg 2011)	**16 to 43%** (Cadroy et al. 2002; Menzel and Hilberg 2011)	NA
Thrombomodulin	**13.5% [9.8%; 17.4%]**	6.0% [− 20.6%; 41.5%]	NA	NA	NA
Von Willebrand Factor	**82.9% [64.1%; 103.8%]**	**166.0% [125.1%; 214.2%]**	NA	NA	NA

Current study: Estimated percentage change due to exercise compared to rest in the placebo group [95% confidence interval of the effect size]. Literature data percentage change due to exercise compared to rest. Figures in bold depict a significant change. NA not available

### Effect of combination of rHuEPO and exercise

The combination of rHuEPO treatment and exercise showed larger changes in several markers compared to exercise alone (in the placebo group). Namely, E-selectin levels post-exercise were 15.3% higher in the rHuEPO treated group than in the placebo group, and 32.1% higher for PF4, see Table 3 and Figs. 1, 3. For the other markers, there was no difference in post-exercise values between the rHuEPO and placebo groups, see Supplemental Table 2.

**Table 3:** Effects of exercise on markers per treatment group

Parameter	Treatment	Raw baseline	EM Day 11	EM Day 25	EM Day 39	EM Day 53	Difference between groups
E-selectin, pg/mL	Placebo	5584 (2104)	5022	5333	5086	4919	15.3% (7.0%, 24.3%) *p* = 0.0004
	rHuEPO	5613 (2001)	5731	5819	5800	6129	
P-selectin, pg/mL	Placebo	9214 (2105)	9789	9719	10280	9211	5.2% (− 2.9%, 13.9%) *p* = 0.2077
	rHuEPO	10300 (1884)	10027	10370	10203	10399	
iCAM, pg/mL	Placebo	339917 (46981)	338125	365918	350488	346019	− 0.9% (− 7.0%, 5.5%) *p* = 0.7677
	rHuEPO	375217 (78950)	342709	357497	337634	349506	
vCAM, pg/mL	Placebo	520833 (76355)	551062	565671	546102	554070	− 3.6% (− 9.2%, 2.4%) *p* = 0.2302
	rHuEPO	566739 (115781)	526734	535286	526161	550035	
Von Willebrand Factor, %	Placebo	164.0 (68.4)	143.4	155.1	161.0	140.8	11.0% (− 1.6%, 25.3%) *p* = 0.0887
	rHuEPO	174.0 (67.3)	174.6	156.8	181.0	154.7	
PF4, pg/mL	Placebo	194624 (116663)	169825	163287	131027	115203	32.1% (4.6%, 66.8%) *p* = 0.0207
	rHuEPO	199970 (148748)	170564	198478	228441	164724	

Raw baseline (and SD) and EM (Estimated Mean) values of coagulation and endothelial activation markers after exercise at the different time points for both treatment groups, including the estimated differences between the treatment groups (95% confidence interval) and *p* value. Data analyzed with a mixed model analysis of variance with fixed factors treatment, time and treatment by time, random factor participant and the pre-value as covariate

## Discussion

Although regular exercise is considered healthy, sporadic cases of exercise-induced thrombotic events, such as ischemic stroke, venous thromboembolism and myocardial infarction have been reported. This is probably related to changes in hemostasis that have been observed after exercise, especially high intensity exercise, possibly in combination with additional predisposing cardiovascular risk factors (Posthuma et al. 2015). One such risk factor could be pharmacological treatment with hematopoietic factors, such as rHuEPO. In anemic patients, rHuEPO increased thrombotic risk, probably in part as a result of hemoconcentration. A meta-analysis of 9353 patients in 57 randomized placebo-controlled trials showed that treatment with erythropoietin was associated with a significantly increased thromboembolic risk [relative risk: 1.67; 95% confidence interval: 1.35 to 2.06] (Bohlius et al. 2006). However, whether rHuEPO also increases thrombotic risk in healthy athletes is unknown. Although the meta-analysis only included studies that used dose levels between 30.000 and 70.000 IU per week, more than allegedly used by athletes (likely more in the range of 10.000 IU per week (Hamilton and Coyle 2012)), this question is relevant given the lack of information on effects in athletes. There is no general consensus on predictive molecular markers for thromboembolic risk, but from a mechanistic point-of-view activation of coagulation pathways, endothelial cells and platelets could ultimately determine thrombotic risk. Therefore, we investigated the effects of rHuEPO doping use on coagulation and endothelial/platelet activation markers, both in rest and after intense exercise, in the same study of which we published the results on the effects of rHuEPO on cycling performance in trained cyclists (Heuberger et al. 2017). The dose regimen in our study is consistent with known practices in professional cycling (Hamilton and Coyle 2012). We quantified molecular markers for activation of the coagulation system and the endothelium after rHuEPO use, exercise, and a combination of both, which may ultimately help us understanding the thrombotic risk of these interventions. This study did not evaluate vascular reactivity from a functional perspective, with a focus on the effect of rHuEPO use and exercise on NO metabolism.

Evaluating the effects of rHuEPO alone, we found an increase in P-selectin and E-selectin, but no clear effects on platelet and endothelial activation by other markers. Exercise alone clearly impacted the hemostatic profile of participants in our study, similar to what has previously been reported (Posthuma et al. 2015) and as shown in Table 2. Our study showed significant changes in all measured markers in the maximal exercise test. Additionally, based on our study and the existing literature, it seems that the effect size on specific markers can depend on the type (duration and intensity) of exercise performed. When combining rHuEPO treatment and exercise, the effects observed on E-selectin were additive, but not synergistic. In addition, PF4 was not significantly increased by rHuEPO alone; however, when rHuEPO and exercise were combined, the increase in PF4 was larger than for exercise alone.

### Clinical relevance

#### Effects on P-selectin

P-selectin is a cellular adhesion molecule enhancing coagulation and activation of leukocyte integrins. P-selectin is mainly released form platelets, but also endothelial cells secrete P-selectin upon activation. Higher P-selectin levels may be an independent risk factor for atherosclerotic disease (Bielinski et al. 2015). In our study, the estimated mean levels of P-selectin after rHuEPO treatment were 9376 pg/mL, an increase of 8% compared to placebo. The exercise test increased P-selectin by 12.2%, but the combination of exercise and rHuEPO did not lead to a significant increase compared to exercise alone. Nevertheless, rHuEPO increased P-selectin levels consistently over the treatment period and to a similar magnitude as the exercise test does.

Though our data clearly demonstrate that both exercise and rHuEPO treatment increased P-selectin levels, the physiological impact of this increase is not clear. Absolute levels of P-selectin seem difficult to compare between studies. Collection should be done in citrated plasma to avoid platelet activation in the tube leading to artificially elevated P-selectin levels (Caine and Blann 2003). However, even when using citrated plasma collection, levels of P-selectin can vary widely between studies (from on average approximately 20000 to 242000 pg/mL in healthy volunteers) (Caine and Blann 2003; Blann et al. 2000; van Poelgeest et al. 2018; Katayama et al. 1993), which might be partly due to differences between assays used (Kappelmayer et al. 2004). So how do the relative increases observed in our study relate to increased risk for clinically relevant thrombotic events? Little is known about the predictive value of P-selectin levels. One study in patients with first unprovoked venous thromboembolism, showed that patients with higher P-selectin levels during the follow-up had a higher risk of recurrence, with 14% higher levels in patients that would experience recurrence (Kyrle et al. 2007). In our study, increases in P-selectin levels were only slightly smaller after rHuEPO treatment and after the exercise test, and more than double after the race (increase of 34.1%). Increases in P-selectin have also been observed after a mild in vivo lipopolysaccharide (LPS) challenge (up to 42% increases) (van Poelgeest et al. 2018). In addition, it is known that P-selectin levels are increased in different cardiovascular and hematological diseases. In thrombotic thrombocytopenic purpura and hemolytic uremic syndrome large differences in P-selectin levels of 274% and 245% with healthy controls were observed (Katayama et al. 1993). In a case–control study deep vein thrombosis was associated with a much smaller difference of 19% (Blann et al. 2000). These findings combined indicate that exercise as well as rHuEPO use potentially are a risk factor for thrombosis.

#### Effects on E-selectin

E-selectin is specifically produced by endothelial cells upon shear stress or P-selectin exposure. Though deep vein thrombosis is not associated with increased systemic levels of E-selectin (Mosevoll et al. 2014), the molecule is a key factor for leukocyte recruitment and the subsequent inflammatory response of the vessel wall, which is key in the thrombotic process. E-selectin inhibition may be a therapeutic modality for thrombosis (Myers et al. 2014). Our study did not evaluate secondary markers for inflammation of the vessel wall but focused on inflammatory biomarkers that are directly related to platelet and endothelial activation. We found that the estimated mean levels of E-selectin after rHuEPO treatment were 5372 pg/mL compared to 4945 pg/mL in the placebo group, an increase of 9%. Exercise increased E-selectin by 7% in the placebo group, but post-exercise E-selectin levels were 15% higher still in the rHuEPO group at 5868 pg/mL, indicating there is an additive effect of rHuEPO and exercise. This leads to a combined increase in E-selectin of approximately 19% versus placebo in rest. Similar to what we describe for P-selectin, absolute baseline values of E-selectin are much smaller than reported previously for healthy volunteers (from 27800 to 54000 pg/mL in a review (Roldan et al. 2003) to 314000 pg/mL in one other study (Raffray et al. 2017)). This review describes significant differences between cases and controls, with increases in E-selectin of 30–50% for diabetes and hypertension. Much larger effects are observed after a mild intravenous in vivo LPS challenge (up to increases of 280%) (van Poelgeest et al. 2018). There was no predictive value of E-selectin for myocardial infarction or death in the follow-up of patients with acute ischemic-type chest pain (Menown et al. 2003). However, patients with coronary artery disease showed higher risk of future death from cardiovascular causes if they had higher levels of iCAM, vCAM and E-selectin, the latter being 29% higher in the group of subjects that experienced events. Most of these reported differences and increases are only approximately twofold higher than the observed effect of the combination of rHuEPO and exercise, and so the combination of rHuEPO and exercise might indicate an increased risk of cardiovascular events compared to exercise alone based on the endothelial marker E-selectin.

#### Other markers

Finally, there is clear evidence that elevated levels of vWF are a predictive factor for ischemic heart disease, cardiovascular mortality and stroke in healthy individuals (Folsom et al. 1999; Jager et al. 1999; Rumley et al. 1999), and iCAM for myocardial infarction (Ridker et al. 1998). In our study, iCAM was unaffected by rHuEPO or the combination of rHuEPO and exercise, however, vWF showed a trend towards an increase after rHuEPO and exercise compared to exercise alone, albeit not significant.

### Mechanism

The origin of E- and P-selectin increments during exercise and particularly upon rHuEPO treatment remains somewhat uncertain. P-selectin is localized in α-granules of platelets, together with other platelet activation markers such as PF4. In addition, P-selectin is found in Weibel-Palade bodies of endothelial cells, together with von Willebrand Factor, so the effect on P-selectin could be mediated both through platelet activation and endothelial activation. However, neither PF4, nor vWF were increased after rHuEPO treatment in rest, contradicting either of these mechanisms (although there was an increase in PF4 after the combination of rHuEPO and exercise). E-selectin on the other hand, is restricted to endothelial cells, but so are iCAM and vCAM, which were not increased after rHuEPO treatment in rest. These increases in E- and P-selectin without changes in other markers are in agreement with previous findings (Heinisch et al. 2012; Stohlawetz et al. 2000). One of these studies showed that E-selectin was already increased 24 h after rHuEPO dosing (Heinisch et al. 2012), indicating an acute mechanism and not an effect mediated through sheer stress by increases in hemoglobin; maturation of normoblasts in bone marrow lasts 5–7 days (McKenzie 1996). Although we did not measure levels in such short timeframe, it is interesting that E-selectin levels were already clearly increased at the first measurement 11 days after start of treatment (Fig. 1), whereas P-selectin levels only started showing increased levels 25 days after start of treatment (Fig. 2). This could indicate these effects are mediated by two different mechanisms. In summary, the mechanism through which rHuEPO increases E- and P-selectin is not completely clarified, and might be through enhancing both endothelial activation and platelet reactivity, as also suggested previously (Stohlawetz et al. 2000).

One limitation to this study is that participants might have been using supplements, other than the study mandated ascorbic acid and ferrous fumarate, that could potentially influence the reported markers. Similarly, diet of participants was not controlled. Concomitant medication other than paracetamol on the other hand was controlled; use was only allowed after consultation with the physician and, therefore, use was limited. Nevertheless, for any of these factors, it is important to note that the randomized design of the study should control for any effects on the outcome measures.

Another study limitation is that we did not evaluate vascular reactivity from a functional perspective, e.g., looking at NO metabolism, or evaluate effects on a broad range of inflammation markers. The focus of this study was on coagulation and platelet and endothelial activation, and therefore, the presented selection of markers was made. With the observed results, it would however be interesting to explore these additional pathways as well.

In conclusion, rHuEPO treatment resulted in endothelial activation in trained cyclists, based on increased levels of P- and E-selectin in rest. Exercise alone increased these markers as well, and the combination with rHuEPO use induced a cumulative effect for E-selectin, but not P-selectin. In addition, exercise induced hypercoagulability as observed by significant changes in all measured markers, including TAT, D-Dimer, aPTT, PF4 and Beta-TG. The combination of exercise and rHuEPO resulted in a larger PF4 response than exercise alone. Based on these markers, exercise potentially increases thrombotic risk, a risk that might be enhanced in combination with additional factors such as rHuEPO use.

## Electronic supplementary material

Below is the link to the electronic supplementary material.

Supplementary material 1 (PDF 175 kb)

## Abbreviations

aPTT: Activated partial thromboplastin time
beta-TG: Beta thromboglobulin
EM: Estimated means
F1 + 2: Prothrombin fragment 1 + 2
FVIII: Factor VIII
Hb: Hemoglobin
Ht: Hematocrit
iCAM: Intercellular adhesion molecule 1
IU: International units
LPS: Lipopolysaccharide
NTR: Nederlands Trial Register (Dutch Trial Registry)
PF4: Platelet factor 4
PT: Prothrombin time
rHuEPO: Recombinant human erythropoietin
TAT: Thrombin:Antithrombin
vCAM: Vascular cell adhesion molecule 1
VO2max: Maximal oxygen uptake
vWF: Von Willebrand Factor antigen
