# Supplementary material for: Additive effect of erythropoietin use on exercise-induced endothelial activation and hypercoagulability in athletes
Source: Eur J Appl Physiol. 2020 Jun 14;120(8):1893–904. doi: 10.1007/s00421-020-04419-0 (PMC7340646; doi:10.1007/s00421-020-04419-0)
Supplement: Supplementary file 1 — Supplementary material 1 (PDF 175 kb) [file 421_2020_4419_MOESM1_ESM.pdf]

SUPPLEMENTAL MATERIAL

Title: Additive effect of erythropoietin use on exercise-induced endothelial activation and hypercoagulability in athletes

Authors: Jules AAC Heuberger[1], Jelle J. Posthuma, Dimitrios Ziagkos, Joris I Rotmans, Johannes MA Daniels, Pim Gal, Frederik E Stuurman, Henri M.H. Spronk, Hugo ten Cate, Jacobus Burggraaf, Matthijs Moerland and Adam F Cohen

Affiliation: [1]Centre for Human Drug Research, Leiden, the Netherlands

Corresponding author: Jules Anne André Cecile Heuberger, PhD (Zernikedreef 8, 2333 CL, Leiden, The Netherlands. E-mail: jheuberger@chdr.nl. Tel: +31 71 5246471. Fax: +31 71 5246499, ORCID: 0000-0001-7202-5088)

Coagulation and endothelial markers

Markers for coagulation were determined in one of two collection tubes: prothrombin time (PT), activated partial thromboplastin time (aPTT), D-dimer and fibrinogen were determined in plasma retrieved from venous blood collected in a 3.2% citrate tube of 2.7 mL, which was processed (spinning at 2000g for 20 minutes) within 30 minutes from collection and stored at ≤ -70 °C. Beta thromboglobulin (beta-TG), prothrombin fragment 1+2 (F1+2), Factor VIII (FVIII), platelet factor 4 (PF4) and Thrombin:Antithrombin (TAT) were determined in plasma retrieved from venous blood collected in a Coagulation Sodium Citrate (CTAD) tube of 3.5 mL, which was pre-cooled in ice-water, and placed back on ice before processing (spinning at 2000g for 20 minutes at 4 °C, brake off) within 30 minutes of collection. Plasma was then pipetted into a pre-cooled tube and spun again for 20 minutes at 2000g and 4 °C, brake off. Plasma was then again pipetted into a pre-cooled tube, mixed, and subdivided in aliquots which were snap frozen with dry ice and methanol for 15 minutes and stored at ≤ -70 °C. For the samples collected just before and directly after the Mont Ventoux race, centrifugation of CTAD tubes was performed at room temperature. Endothelial function markers E-selectin, intercellular adhesion molecule 1 (iCAM), P-selectin, thrombomodulin, vascular cell adhesion molecule 1 (vCAM) and Von Willebrand Factor antigen (vWF) were measured from the same CTAD tube.

These markers were determined as follows: Activated partial thromboplastin time, Prothrombin time and Fibrinogen were determined using the STA-Evolution Coagulation analyzer (Diagnostica Stago C1-030, Theale, England), D-dimer was determined using the STA-R MAX coagulation analyzer (Diagnostica Stago, Theale, England). Enzyme-linked immunosorbent assay (ELISA) was used for the quantitative detection of: Human CXCL7 (for Beta Thromboglobuline) PicoKine™ ELISA Kit (Boster Biological Technology, Pleasanton CA, USA, Catalog # EK0729), Human sE-Selectin PicoKine™ ELISA Kit (Boster Biological Technology, Pleasanton CA, USA, Catalog # EK0501), Human P-Selectin PicoKine™ ELISA Kit (Boster Biological Technology, Pleasanton CA, USA, Catalog # EK0505), Human PF4/CXCL4 PicoKine™ ELISA Kit (Boster Biological Technology, Pleasanton CA, USA, Catalog # EK0726), human thrombomodulin PicoKine™ ELISA Kit (Boster Biological Technology, Pleasanton CA, USA, Catalog # EK0917). vWF was measured with an in-house developed validated ELISA assay. The vWF ELISA was calibrated against the WHO standard plasma for vWF and has a total assay CV of 8%. Prothrombin activation F1+2 and TAT levels were determined using the Enzygnost (monoclonal) ELISA kit (Siemens Healthcare Diagnostics, Siemens NL). All assays were performed according to the manufacturers' protocol.

FVIII activity was measured by the one-stage clotting assay on a Siemens BCS-XP analyzer (Siemens Healthcare Diagnostics) with the use of commercial reagents (Siemens Actin FSL, Siemens Healthcare Diagnostics) with calibration against a normal reference plasma (SHP, Siemens Healthcare) calibrated against the WHO standard plasma for factor FVIII. iCAM and vCAM were measured using MesoScale Discovery's V-PLEX kits.

Maximal versus submaximal exercise effects

The effects on coagulation and endothelial markers of an exercise test and a race can also be compared in this study. All markers were significantly affected by the exercise test, where this was only the case for half the markers after the race. As the analysis for the race is based on one measurement compared to five for the exercise test, it could be that some effects of the race remained undetected due to the lower power (e.g. for E-selectin, iCAM and vCAM, see Table 3). There are however, also clear differences in effects on haemostatic profile between the two exercise types: PT is decreased in exercise test, but does not seem affected by a longer submaximal exercise such as the race. Fibrinogen, D-dimer and F1+2 are increased after exercise test, but were not affected by the race. Also, TAT was increased to a greater extent after exercise test than after the race (see also Figure 3). On the other hand, the race increased P-selectin and vWF more than the exercise test did (see also Figure 4). Effects on aPTT, beta TG, FVIII and PF4 are similar for both exercise types. Our observed effects of exercise on haemostatic profile are similar to those reported in a previously published review.[3] Platelet count increased with 10.6% after the race, somewhat smaller than the already reported 25-27% for short (sub-)maximal exercise, but very similar to what has been observed for the more comparable long (>60min) submaximal exercise (12%). Most remarkable differences with literature were observed for F1+2, FVIII and TAT which all showed much higher increases than previously reported (2-4-fold higher).

Supplemental Table 1 Effects of rHuEPO on markers in rest

Parameter	Treatment	Raw baseline	EM Day 11	EM Day 14	EM Day 25	EM Day 28	EM Day 39	EM Day 42	EM Day 53	EM Pre race	Difference between groups
Hemoglobin (mmol/L)	Placebo	8.9 (0.5)	*ND*	8.8	*ND*	8.7	*ND*	8.9	*ND*	9.4	0.60 (0.44, 0.77) p=<0.0001
	rHuEPO	9.0 (0.5)	*ND*	9.2	*ND*	9.4	*ND*	9.6	*ND*	10.1	
Hematocrit (L/L)	Placebo	0.431 (0.022)	*ND*	0.437	*ND*	0.436	*ND*	0.438	*ND*	0.460	0.0330 (0.0250, 0.0409) p=<0.0001
	rHuEPO	0.433 (0.022)	*ND*	0.458	*ND*	0.470	*ND*	0.474	*ND*	0.499	
Platelet count, (*10E^{9})/L	Placebo	226 (44)	*ND*	217	*ND*	221	*ND*	217	*ND*	210	-0.1% (-7.1%, 7.3%) p=0.97
	rHuEPO	228 (52)	*ND*	223	*ND*	229	*ND*	219	*ND*	195	
Activated partial thromboplastin time, s	Placebo	30.7 (1.4)	30.1	30.1	29.8	30.0	30.4	29.6	30.4	30.0	1.5% (-0.3%, 3.4%) p=0.097
	rHuEPO	29.9 (1.9)	29.8	30.4	30.4	30.4	31.0	30.4	31.1	30.6	
Prothrombin time, s	Placebo	14.5 (0.6)	14.5	14.3	14.5	14.5	14.6	14.3	14.3	14.2	-0.0% (-1.6%, 1.6%) p=1.00
	rHuEPO	14.4 (0.9)	14.5	14.4	14.7	14.3	14.4	14.4	14.4	14.0	
Fibrinogen, g/L	Placebo	2.6 (0.3)	2.4	2.5	2.5	2.5	2.4	2.4	2.5	2.5	1.3% (-3.9%, 6.8%) p=0.62
	rHuEPO	2.7 (0.4)	2.5	2.5	2.4	2.6	2.4	2.5	2.5	2.5	
D-dimer, ng/mL	Placebo	231.6 (145.1)	253.4	222.3	213.1	217.6	186.8	217.1	205.5	251.8	-1.3% (-17.0%, 17.4%) p=0.88
	rHuEPO	258.5 (157.3)	217.1	216.4	242.8	204.9	190.8	218.6	221.4	228.9	
Beta Thromboglobulin, pg/mL	Placebo	16113 (8283)	26836	11777	22008	14520	15088	16944	15554	23881	11.2% (-10.0%, 37.6%) p=0.32
	rHuEPO	31194 (85294)	26350	13585	27421	14135	19539	16419	19785	25535	
Prothrombin fragment 1+2, pmol/L	Placebo	118.6 (53.4)	169.4	102.9	167.2	108.1	116.4	134.5	122.6	137.1	-0.5% (-15.9%, 17.8%) p=0.95
	rHuEPO	368.2 (1262.8)	160.5	106.1	160.8	109.9	127.5	125.5	126.5	131.1	

Parameter	Treatment	Raw baseline	EM Day 11	EM Day 14	EM Day 25	EM Day 28	EM Day 39	EM Day 42	EM Day 53	EM Pre race	Difference between groups
Factor VIII, %	Placebo	129 (37)	119	120	129	129	125	119	117	130	3.7% (-3.1%, 10.9%) p=0.29
	rHuEPO	137 (43)	129	123	136	132	120	125	128	131	
Thrombin:Antithrombin, ng/mL	Placebo	2.9 (3.5)	4.3	1.5	3.7	1.6	1.9	2.0	1.8	1.9	4.1% (-20.0%, 35.5%) p=0.76
	rHuEPO	14.7 (64.0)	3.1	1.5	4.4	1.6	2.1	1.9	2.2	2.3	
Thrombomodulin, pg/mL	Placebo	1383 (1358)	1323	1347	1320	1368	1322	1328	1364	1385	-2.8% (-10.2%, 5.2%) p=0.48
	rHuEPO	1936 (2756)	1252	1283	1218	1317	1319	1315	1387	1377	

Raw baseline (and SD) and EM (Estimated Mean) values of hematological, coagulation and endothelial function markers in rest at the different time points for both treatment groups, including the estimated differences between the treatment groups (95% confidence interval) and P-value. Data analyzed with a mixed model analysis of variance with fixed factors treatment, time and treatment by time, random factor participant and the pre-value as covariate. *ND*: not determined at this timepoint.

Supplemental Table 2 Effects of exercise on markers per treatment group

**Parameter**	**Treatment**	**Raw baseline**	**EM Day 11**	**EM Day 25**	**EM Day 39**	**EM Day 53**	**Difference between groups**
Activated partial thromboplastin time, s	Placebo	29.1 (11.9)	27.3	27.0	27.2	27.7	-0.3% (-3.2%, 2.7%) 0.8380
	rHuEPO	26.2 (8.2)	27.0	27.3	26.5	28.0	
Prothrombin time, s	Placebo	14.2 (0.7)	14.3	14.4	14.1	14.0	-0.5% (-2.4%, 1.5%) 0.6379
	rHuEPO	14.3 (1.0)	14.1	14.4	14.1	13.9	
Fibrinogen, g/L	Placebo	2.8 (0.4)	2.5	2.6	2.6	2.7	3.3% (-3.2%, 10.3%) 0.3223
	rHuEPO	2.7 (0.4)	2.7	2.5	2.7	2.9	
D-dimer, ng/mL	Placebo	465.5 (370.6)	375.8	420.40	234.3	249.0	17.9% (-7.6%, 50.4%) 0.1803
	rHuEPO	1793.4 (4383.1)	366.7	435.7	428.8	259.8	
Beta Thromboglobulin, pg/mL	Placebo	43069 (23902)	62389	43455	36049	32754	17.9% (-10.7%, 55.6%) 0.2393
	rHuEPO	125259 (231695)	45578	64846	54561	38299	
Prothrombin fragment 1+2, pmol/L	Placebo	331.3 (179.6)	344.0	287.7	244.9	218.6	15.5% (-6.4%, 42.4%) 0.1736
	rHuEPO	713.3 (1343.7)	302.4	402.9	334.1	231.4	
Factor VIII, %	Placebo	314 (115)	255	291	281	262	8.7% (-4.6%, 24.0%) 0.2038
	rHuEPO	287 (100)	284	293	328	279	
Thrombin:Antithrombin, ng/mL	Placebo	30.0 (21.6)	27.9	19.5	15.3	13.2	12.0% (-17.1%, 51.3%) 0.4520
	rHuEPO	52.2 (79.5)	19.8	26.4	26.0	12.7	

Parameter	Treatment	Raw baseline	EM Day 11	EM Day 25	EM Day 39	EM Day 53	Difference between groups
Thrombomodulin, pg/mL	Placebo	1518 (1349)	1375	1459	1488	1560	-2.1%<br>(-10.2%, 6.6%)<br>0.6129
	rHuEPO	2016 (2958)	1354	1358	1514	1533	

Raw baseline (and SD) and EM (Estimated Mean) values of coagulation and endothelial function markers after exercise at the different time points for both treatment groups, including the estimated differences between the treatment groups (95% confidence interval) and P-value. Data analyzed with a mixed model analysis of variance with fixed factors treatment, time and treatment by time, random factor participant and the pre-value as covariate.
